# The Male Staff of Hospitals

**Published:** 1912-01-27

**Authors:** 


THE MALE STAFF OF HOSPITALS.

The Secretary of the Norfolk and Norwich Hospital and the Secretary of the Royal Berkshire Hospital are thanked for their letters containing information as to the number of the male staff of their hospitals. Will hospital secretaries in general kindly supply us with similar information, giving the number and composition of the male staff and the numbers already insured. Such information must, in its completed form, materially help each hospital when they have to determine the best course to take in regard to the National Insurance Act.

